# Colony Foundation in an Oceanic Seabird

**DOI:** 10.1371/journal.pone.0147222

**Published:** 2016-02-24

**Authors:** Ignacio Munilla, Meritxell Genovart, Vitor H. Paiva, Alberto Velando

**Affiliations:** 1 Departamento de Botánica, Facultade de Bioloxía, Universidade de Santiago de Compostela, Santiago de Compostela, Galicia, Spain; 2 Marine and Environmental Sciences Centre, Department of Life Sciences, University of Coimbra, Coimbra, Portugal; 3 Departamento de Ecoloxía e Bioloxía Animal, Universidade de Vigo, Vigo, Galicia, Spain; CEFE, FRANCE

## Abstract

Seabirds are colonial vertebrates that despite their great potential for long-range dispersal and colonization are reluctant to establish in novel locations, often recruiting close to their natal colony. The foundation of colonies is therefore a rare event in most seabird species and little is known about the colonization process in this group. The Cory’s shearwater (*Calonectris diomedea*) is a pelagic seabird that has recently established three new colonies in Galicia (NE Atlantic) thus expanding its distribution range 500 km northwards. This study aimed to describe the establishment and early progress of the new Galician populations and to determine the genetic and morphometric characteristics of the individuals participating in these foundation events. Using 10 microsatellite loci, we tested the predictions supported by different seabird colonization models. Possibly three groups of non-breeders, adding up to around 200 birds, started visiting the Galician colonies in the mid 2000’s and some of them eventually laid eggs and reproduced, thus establishing new breeding colonies. The Galician populations showed a high genetic diversity and a frequency of private alleles similar to or even higher than some of the large historical populations. Most individuals were assigned to several Atlantic populations and a few (if any) to Mediterranean colonies. Our study suggests that a large and admixed population is settling in Galicia, in agreement with predictions from island metapopulation models of colonization. Multiple source colonies imply that some birds colonizing Galicia were dispersing from very distant colonies (> 1500 km). Long-distance colonizations undertaken by relatively large and admixed groups of colonizers can help to explain the low levels of genetic structure over vast areas that are characteristic of most oceanic seabird species.

## Introduction

Colonization, defined as the process leading to the establishment of a population in a novel location, is essential for the persistence of species, especially in a changing world [1] where range shifts commonly occur in response to climate change [2]. In many organisms, colonizations are essential to the dynamics of spatially structured populations [3] which are governed by local extinctions and the colonization of empty habitat patches [4]. In social species, different colonization models have different consequences for the genetic composition and structure of the newly established populations [5–7]. Intraspecific genetic admixture is likely to contribute positively to the fitness and the adaptive potential of founder groups, thus enhancing their chances of persistence, especially in novel habitats [8]. By contrast, serial founder effects and population bottlenecks -as in one dimensional stepwise colonizations- are expected to promote inbreeding, reduced genetic variability and loss of private alleles in populations [9, 10]. Thus, successful colonization, (i.e. the persistence of a new population over time) depends on intrinsic factors, such as the size and the composition of the founder group [11].

An analysis of historical species introductions has suggested that in birds the probability of establishment increases with the number of founders [12]. Importantly, the demographic and genetic components of propagule pressure are interrelated [13]. Small populations are prone to extinction because they are disproportionately affected by genetic and demographic stochasticity, a notion that relates to the minimum viable population size [14]. Studies at the onset of colonization are quite rare in vertebrates, which implies that our knowledge of founder vertebrate populations is based on inferences made from populations already established. Therefore, the individual characteristics of founders and the actual timing of the founding event, including estimates of population sizes and data on breeding output over the first few years are rarely known from first hand. A few exceptions to this come from studies on the establishment of some seabird colonies [15–18] and subcolonies [19].

The great majority of seabird species breed colonially (98%; [20]). Hence their populations are distributed in local patches (colonies) that are considered analogous to local populations in metapopulation dynamics [21–24]. For settlement decisions, seabirds can rely on personal and social information collected during prospecting such as presence and breeding performance of conspecifics [25–27]. In new habitat patches, however, the only type of information available to individuals is based on their direct interaction with the environment, and founders must overcome an “information barrier” (*sensu* Forbes and Kaiser [28]) and have to deal with greater uncertainty [29–31]. Thus, despite their great potential for long-range dispersal and colonization, seabirds are reluctant to establish in novel locations, mostly recruiting close to their natal colony [32–34]. The foundation of colonies is a rare event in most seabird species and little is known about the colonization process in this group.

**Fig 1:**
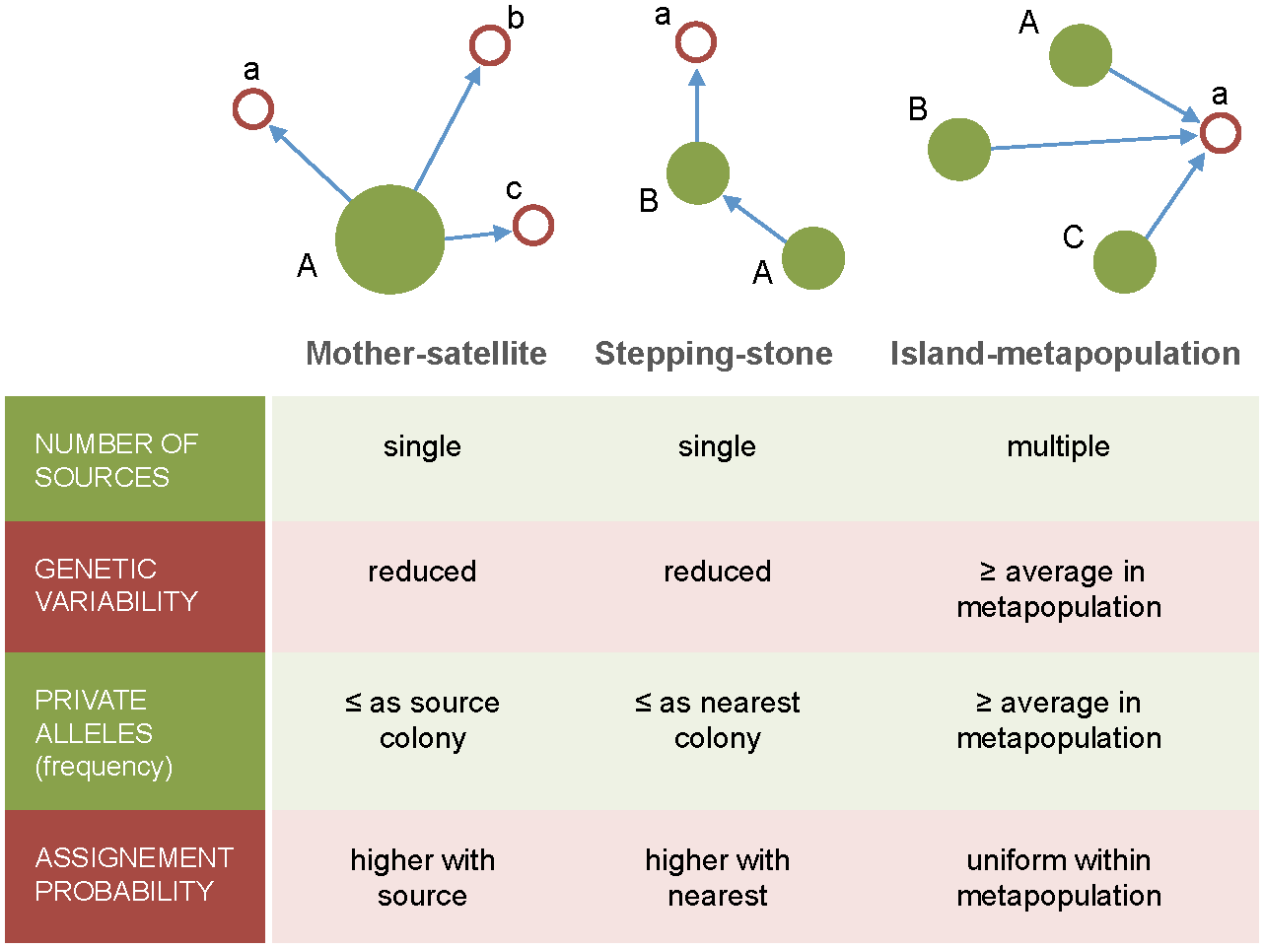
Colonization models proposed for seabirds in the literature: Mother-satellite, Stepping-stone and Island-metapopulation. The diagrams show source (upper case) and newly founded (lower case) colonies with arrows indicating the flow of colonizers. The models support different predictions in terms of the genetic variability and private alleles (i.e. alleles that are not found in other sampled populations) of the founder populations as shown respectively in the lower section.

Several models have been proposed in the seabird literature to explain the formation of new colonies (Fig 1): (i) Through a mother-satellite model, where new colonies are founded by individuals from a single, large and highly productive mother colony, that establish at the verge of its foraging range, like the expansion of the great cormorant *Phalacrocorax carbo* over Denmark and the Netherlands [35]. (ii) Through stepping stone models of expansion, in which new colonies are founded by individuals from the nearest existing colony, and thus we expect to find a reduced number of source populations and an isolation by distance pattern, as suggested for the expansion of the Northern fulmar *Fulmarus glacialis* across Britain [36, 37]. (iii) Through island metapopulation models, where multiple source colonies contribute to newly colonized sites and we thus expect high levels of genetic admixture in the new colonies, as has been suggested for the Laysan albatross *Phoebastria immutabilis* in the North Pacific Ocean [38] and the kittiwake *Rissa tridactyla* in Eastern Canada [17].

Our study model, the Cory´s shearwater (*Calonectris diomedea*), is the largest of procellariids in the North Atlantic (see [39] for a thoroughly revision of the species´ biology) and breeds in the Macaronesian and Mediterranean islands. Cory’s shearwaters are highly philopatric and show a high degree of site tenacity [40, 41]. The Atlantic and the Mediterranean populations are considered as distinct taxonomic units; however whether they should be considered at the species (*C*. *borealis* and *C*. *diomedea* respectively) [42, 43] or subspecies level (*C*. *d*. *borealis* and *C*. *d*. *diomedea*) [44] is currently open to debate. Cory´s shearwaters are strong flyers, with some individuals travelling hundreds or even thousands of kilometers on a single foraging trip [45]. Several studies have suggested that the spatial structure of Cory´s populations over the Atlantic and the Mediterranean is particularly complex and shaped by rare albeit recurrent long-distance dispersal events [44], but virtually nothing is known about their colonization patterns and mechanisms. In 2007–2008 three new Cory´s shearwater colonies (Cíes, Sisargas and Coelleira) were discovered in the coasts of Galicia (NW Iberia) [46], thus extending the breeding range of the species in the Atlantic 500 km northwards (ca. 5°). It is unlikely that any of these colonies were started before the mid 2000’s, especially in Cíes and Sisargas, as these islands have been intensively surveyed for other seabirds since the mid 1990’s. Therefore, the onset of the Galician colonies provided the opportunity to study the process of colony foundation at the leading edge of the species range.

In this study we aimed at: (i) describing the establishment and early progress of the new Cory´s shearwater colonies in Galicia; (ii) analyzing the genetic diversity and the number of alleles private to these new populations using microsatellites as genetic markers; (iii) determining the most likely sources (geographic origin) of the individuals participating in these founding events by drawing evidence from genetic markers and morphological characteristics. We were particularly interested in testing which of the three models of colony formation best fit our observations, as they are expected to produce contrasting patterns in the genetic diversity and the number of private alleles in the new populations (Fig 1).

## Results

### Establishment and development of new Cory´s shearwater colonies in Galicia

**Fig 2:**
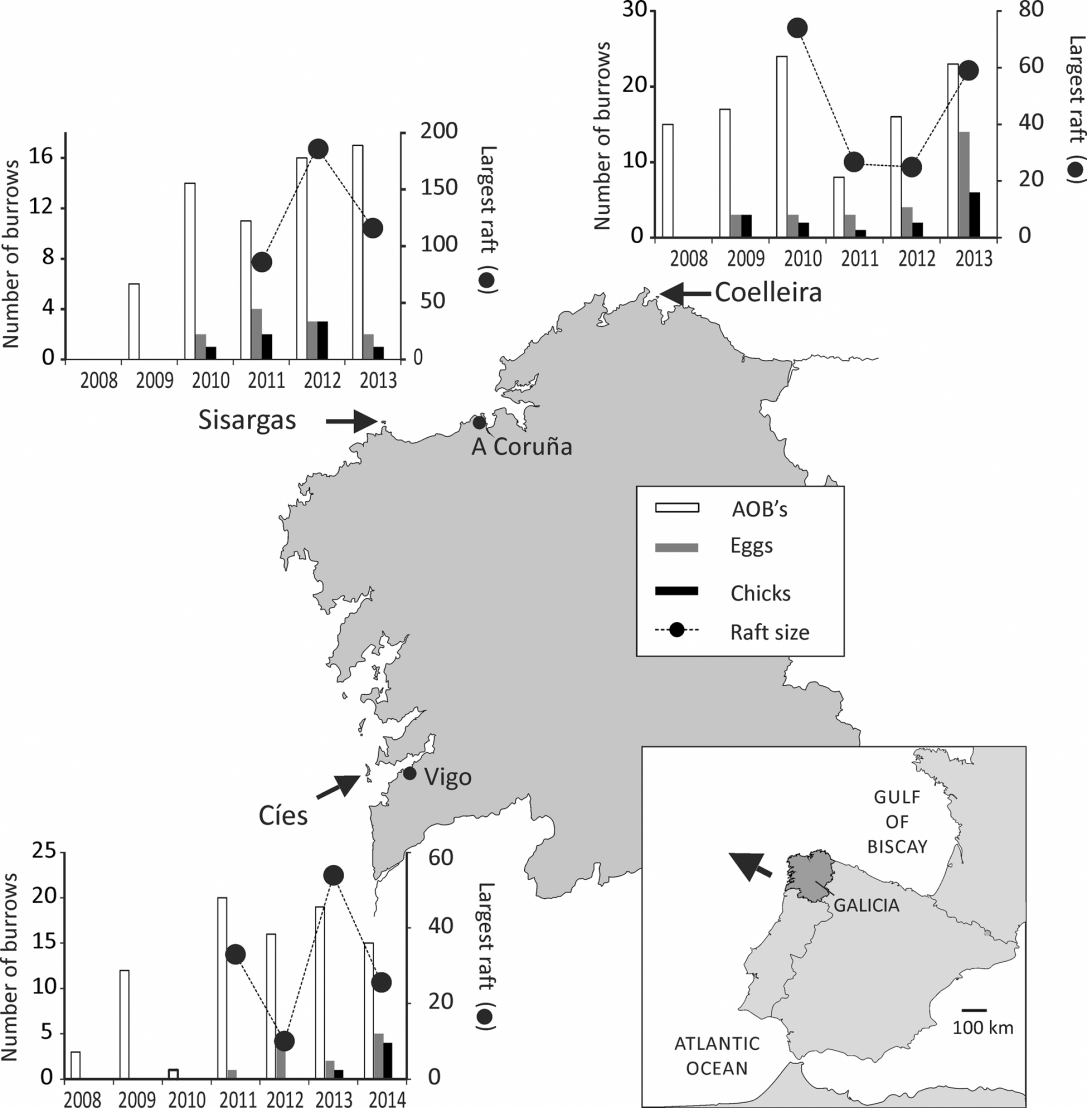
Location and development of the newly established colonies of Cory’s shearwaters in Galicia. The bar charts show the number of apparently occupied burrows (AOB’s; open bars) and the number of burrows containing eggs (dark grey bars) and chicks (closed bars), in each of the newly established breeding colonies of Cory’s shearwater in Galicia (Coelleira, Sisargas and Cíes) during 2008–2014. The closed circles represent the size of the largest raft observed assembling near the colony during the main breeding season (May-August).

We made 168 captures of 128 different individuals, including 9 chicks. A bird observed inside a burrow in Cíes in May 2007 is the first known record indicative of the breeding of Cory´s shearwaters in NW Iberia. Later in that year a few birds were reported prospecting in Coelleira and by 2011, three small colonies and a few scattered burrows were active in three insular localities: Cíes, Sisargas and Coelleira (Fig 2). A total of 86 apparently occupied burrows (AOB´s) were marked over the study period (27 in Cíes, 21 in Sisargas and 38 in Coelleira) and by 2013 we estimated that at least 61 burrows were occupied (21 Cíes, 17 Sisargas, 23 Coelleira).

In any given year, the occupation of burrows varied from full occupation (successful breeders) to sporadic occupation by a single prospector. Overall, the number of AOB´s increased from 24 to 61, albeit in the first years only a few burrows were occupied by laying pairs. The proportion of active burrows occupied by breeders and the number of chicks produced increased over the study period both in Coelleira and Cíes (Fig 2). Thus, in the last year of the study period as much as 61% of active burrows were occupied in Coelleira (14 laying pairs producing 6 chicks) and 36% in Cíes (5 laying pairs producing 4 chicks). In Sisargas the number of breeding pairs remained low throughout the study period. Predation by introduced terrestrial carnivores was observed in Sisargas and possibly affected the early development of the Cíes population. In Sisargas we found 23 adults likely killed by feral cats (*Felis catus*) in 2010. In Cíes, American mink (*Neovison vison*) predated on European shags nesting close to the area where the shearwaters first established in 2009 and 2010.

The number of birds assembling in rafts near the colony fluctuated somewhat from year to year in the three colonies but without a clear trend (Fig 2). The largest rafts observed throughout the study period comprised 74 individuals in Coelleira (2010), 187 in Sisargas (2012) and 54 in Cíes (2013). The size of the rafts varied significantly among colonies (F_2,14_ = 14.75; P < 0.001) and did not vary significantly across years (F_1,15_ = 0.986; P = 0.336) or months (F_1,15_ = 0.173; P = 0.683).

### Genetic diversity of the founder populations

The genetic statistics showed that the diversity of the new Galician populations was high and within the range of values of potential source populations (Table 1). Notably, two of the Galician populations showed the highest values of mean number of alleles per locus.

**Table 1:** Indices of genetic diversity in Cory’s shearwater populations from Galicia (new colonies), the Atlantic and the Mediterranean.

Area	Colony	N	P	*a*	H_o_ (±SD)	H_e_ (±SD)	Gene diversity	F_IS_
**Galicia**	*Coelleira*	*55*	*9/9*	*5*.*9*	*0*.*42±0*.*23*	*0*.*44±0*.*26*	*0*.*43±0*.*24*	*0*.*14*
	*Sisargas*	*27*	*8/8*	*5*.*6*	*0*.*47±0*.*26*	*0*.*55±0*.*26*	*0*.*43±0*.*24*	*0*.*15*
	*Cíes*	*21*	*8/9*	*5*.*1*	*0*.*43±0*.*25*	*0*.*56±0*.*24*	*0*.*50±0*.*27*	*0*.*23*
**Atlantic**	*Berlenga*	*20*	*9/9*	*4*.*3*	*0*.*36±0*.*29*	*0*.*44±0*.*28*	*0*.*44±0*.*25*	*0*.*21*
	*Canarias*	*25*	*10/9*	*4*.*8*	*0*.*40±0*.*25*	*0*.*49±0*.*24*	*0*.*44±0*.*25*	*0*.*20*
	*Selvagens*	*18*	*9/8*	*5*.*0*	*0*.*54±0*.*27*	*0*.*61±0*.*24*	*0*.*54±0*.*30*	*0*.*11*
	*Desertas*	*20*	*10/9*	*5*.*3*	*0*.*42±0*.*26*	*0*.*52±0*.*24*	*0*.*47±0*.*26*	*0*.*21*
	*Azores*	*20*	*10/10*	*4*.*4*	*0*.*42±0*.*27*	*0*.*44±0*.*29*	*0*.*43±0*.*24*	*0*.*06*
**Mediterranean**	*Aire*	*22*	*10/9*	*4*.*9*	*0*.*46±0*.*22*	*0*.*57±0*.*21*	*0*.*52±0*.*28*	*0*.*19*
	*Pantaleu*	*28*	*10/10*	*4*.*5*	*0*.*39±0*.*23*	*0*.*51±0*.*19*	*0*.*50±0*.*27*	*0*.*24*
	*Habibas*	*19*	*9/9*	*4*.*2*	*0*.*41±0*.*29*	*0*.*52±0*.*29*	*0*.*48±0*.*27*	*0*.*22*

N = sample size (individuals); P = number of suitable loci (< 5% missing data)/ number of polymorphic loci; *a* = mean number of alleles per locus; H_o_ and H_e_ = observed and expected heterozygosity; overall mean gene diversity; F_IS =_ estimates for the inbreeding coefficient.

**Fig 3:**
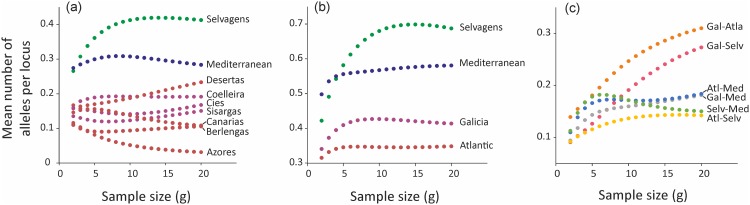
Private alleles. Mean number of private alleles per locus (i.e. alleles that are not found in other sampled populations) as functions of standardized sample size (g). Private alleles were analyzed using: (a) individual Atlantic colonies and the Mediterranean group (Pantaleu, Aire and Habibas); (b) four combination of colonies, Galicia (Coelleira, Sisargas and Cíes), Atlantic (Azores, Desertas, Canarias and Berlengas), Selvagens and Mediterranean (see Fig 4 for rationale underlying this grouping); (c) private alleles of all pair combinations of the four major breeding areas. Private allelic richness for a pair combination estimates the number of distinct alleles private to a group of populations and found in all populations in the group, thus indicative of shared ancestry.

The estimated number of private alleles per locus (i.e. alleles exclusively found in a population) as evaluated by the rarefaction method implemented in the computer program ADZE indicated that Selvagens possessed the highest number of private alleles, and that Azores showed the smallest values (Fig 3a). The values of the Galician colonies were intermediate between these two, and larger than Canarias and Berlenga, the nearest colonies. When private alleles were examined in sets of colonies according to major breeding areas, the analysis indicated higher allelic richness in Galicia compared to the Atlantic group without Selvagens (Fig 3b). Additionally, we also applied the rarefaction approach for counting alleles private to various pair combinations of the major breeding areas. The combination Galicia-Atlantic and Galicia-Selvagens showed the highest number of alleles private to combinations of two geographic regions, whereas the smallest values occurred in the Atlantic-Selvagens combination (Fig 3c). This result is consistent with shared ancestry between Galicia and both Atlantic and Selvagens colonies (Fig 3b).

### Population structure

Pairwise *F*ST values ranged from 0.015 to 0.246 (0.017–0.034 in Galicia) with an average *F*ST = 0.109 (CV = 1.42). These values represent moderate levels of population differentiation (Table 2). After the Bonferroni correction 36 out of 55 comparisons (65%) were significant. The comparisons between the three Galician colonies produced only one significant albeit low pairwise *F*ST value thus likely indicating a common pool of source populations. Overall, the results of the Mantel test did not support a significant correlation between genetic and geographic distances, either with (*r*_*m*_ = 0.089; P = 0.282; n = 12) or without (*r*_*m*_ = 0.133; P = 0.237; n = 9) the Galician colonies; the correlation was however, significant for the subset of historical Atlantic colonies (*r*_*m*_ = -0.613; P = 0.033; n = 5).

**Table 2:** Pairwise *F*_ST_ values for microsatellites of Cory´s shearwaters (above diagonal) and distances in km (below diagonal) from 11 breeding localities in the Atlantic and the western Mediterranean.

	Atlantic								Mediterranean		
	Cíes	Sisargas	Coelleira	Berlenga	Azores	Canarias	Selvagens	Desertas	Pantaleu	Aire	Habibas
**Cíes**	*-*	*0*.*017*	*0*.*022*	*0*.*023*	*0*.*024*	*0*.*026*	***0*.*149***	***0*.*184***	***0*.*247***	***0*.*071***	*0*.*017*
**Sisargas**	*158*	*-*	***0*.*034***	*0*.*028*	*0*.*026*	*0*.*029*	***0*.*151***	***0*.*181***	***0*.*241***	***0*.*085***	*0*.*024*
**Coelleira**	*270*	*115*	*-*	***0*.*029***	*0*.*016*	***0*.*032***	***0*.*158***	***0*.*195***	***0*.*245***	***0*.*063***	***0*.*025***
**Berlenga**	*318*	*460*	*580*	*-*	*0*.*034*	*0*.*029*	***0*.*121***	***0*.*169***	***0*.*198***	***0*.*077***	*0*.*023*
**Azores**	*1515*	*1550*	*1680*	*1425*	*-*	*0*.*027*	***0*.*145***	***0*.*183***	***0*.*230***	***0*.*055***	*0*.*028*
**Canarias**	*1670*	*1795*	*1925*	*1400*	*1390*	*-*	***0*.*150***	***0*.*220***	***0*.*246***	***0*.*086***	***0*.*031***
**Selvagens**	*1470*	*1590*	*1735*	*1180*	*1235*	*210*	*-*	*0*.*119*	*0*.*027*	***0*.*133***	***0*.*147***
**Desertas**	*1260*	*1375*	*1510*	*1000*	*1021*	*475*	*280*	*-*	*0*.*141*	***0*.*191***	***0*.*186***
**Pantaleu**	*1770*	*1940*	*2040*	*1435*	*2640*	*2150*	*1995*	*1950*	*-*	***0*.*198***	***0*.*231***
**Aire**	*1915*	*2090*	*2180*	*1520*	*2785*	*2300*	*2150*	*2060*	*186*	*-*	***0*.*070***
**Habibas**	*1340*	*1500*	*1604*	*935*	*2200*	*1700*	*1960*	*1480*	*520*	*655*	***-***

Statistically significant *F*_ST_ values after Bonferroni correction (P<0.005) are shown in bold. Newly founded populations (Cíes, Sisargas and Coelleira) are shown for comparison as they have a structure that likely reflects the sources of founding individuals. The vertical line separates Atlantic and Mediterranean colonies.

**Fig 4:**
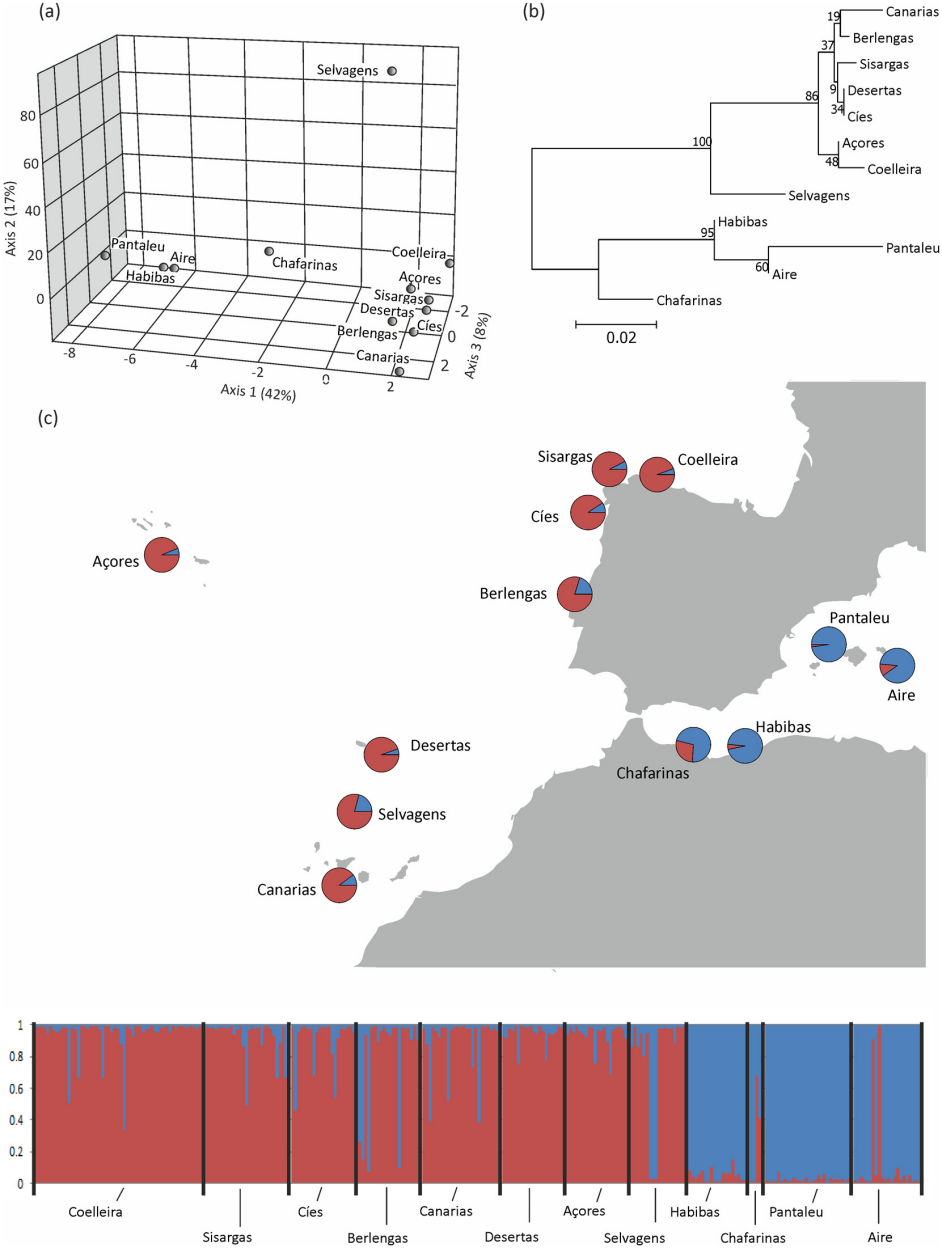
Population genetic structure of Cory’s shearwater. (a) Factorial correspondence analysis performed on pairwise allele frequency differences using GENETIX v.4. (b) Population-level neighbour-joining tree based on *F*_*ST*_ across the 10 loci with per cent bootstrap support (10,000 replicates) shown at nodes. (c) Inferred ancestry (Q-matrix) of the two genetic clusters (red: Atlantic cluster; blue: Mediterranean cluster) for populations (upper panel) and individuals (lower panel) estimated by permutation from ten Structure analyses for individuals and populations, respectively, using Clumpp.

The first axis of the factorial correspondence analysis (42% of variance) clearly split Atlantic and Mediterranean populations, whereas the second axis (17% of variance) separate Selvagens from the rest (Fig 4a). The old and new (Galician) Atlantic populations ordered along the third axis (8% of variance). Our population-level tree also separated Mediterranean and Atlantic populations with very high bootstrap support (100%). In the Atlantic node, the support for the node separating Selvagens from other Atlantic populations was relatively high (86%), but our analysis revealed low support for the remaining nodes among Atlantic populations (<50%). Galician populations clustered into the core Atlantic clade (Fig 4b).

The Bayesian estimates of population structure corroborated the separation between Mediterranean and Atlantic populations (Fig 4c). The model-based clustering method implemented in STRUCTURE suggested that the model with two genetic clusters (*K* = 2) was considerably better than alternate models. These two clusters broadly corresponded to the main geographical areas, Atlantic and Mediterranean. Mean membership coefficients (*Q*) for all populations into one of these two demes were high (mean maximum *Q* = 0.88 ± 0.089, n = 12), with Selvagens (0.76) and Chafarinas (0.68) as lowest (Fig 4c). Mean membership coefficients into the Atlantic deme for the individuals captured at the Galician colonies were generally high (mean Atlantic *Q* 0.93 ± 0.012, n = 103). Four Galician individuals showed mixed or Mediterranean origin (Atlantic *Q* range: 0.33–0.66), but similar patterns of admixture were found in other Atlantic populations (Fig 4).

### Assignment of founders

**Fig 5:**
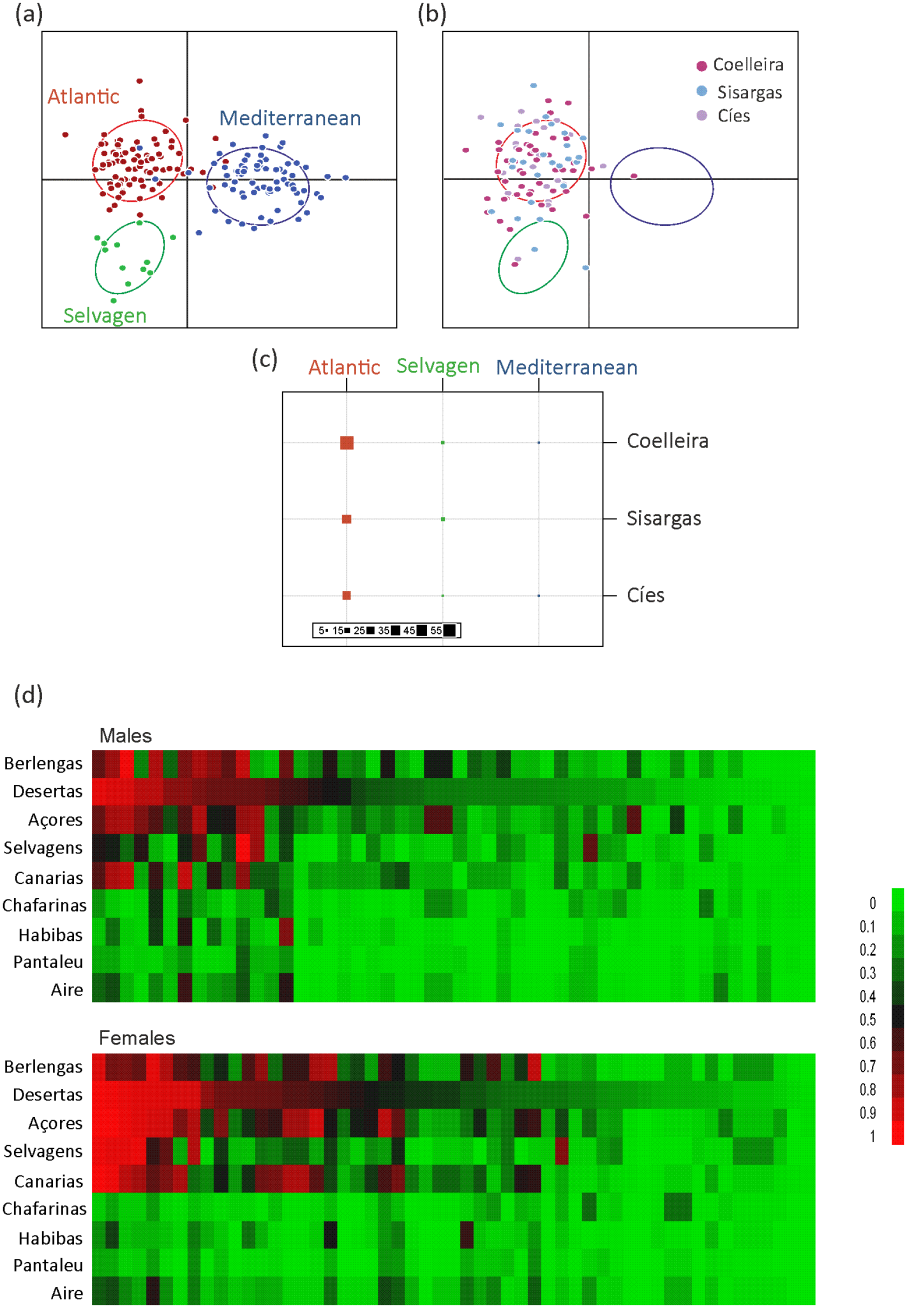
Assignment of Galician individuals to potential source populations. (a-c) Discriminant Analysis of Principal Components (DAPC in adegenet) on Cory’s shearwater populations using three main reference areas (Core Atlantic, Selvagens and Mediterranean): (a) Scatter plot of individuals used as reference and groups as inertia ellipses according with two discriminant functions, representing 93% of variance (x-axis: first discriminant function [DF1], 66% of variance, y-axis: second discriminant function [DF2], 27% of variance); (b) Scatter plot of individuals from the three Galician colonies; (c) Number of individuals assigned to the main reference areas; (d) Heat plot of assignment probabilities of Cory’s shearwaters captured at the newly established Galician colonies. Each column represents an individual (50 females and 53 males) and colour the probability to be assigned to a reference population as estimated by GeneClass2. Probability was calculated independently for each population by Monte-Carlo resampling.

A discriminant analysis of principal components (DAPC) using all source populations had low discriminant power as only 65% of individuals were correctly reclassified. Interestingly, all the individuals from Selvagens classified correctly, but in the other populations the proportion of correct reassignments was low (62%). However, when we used a DAPC with three main reference areas (Core Atlantic, Selvagens and Mediterranean), 96% of individuals were correctly assigned to their actual main breeding area (Fig 5a). According to the DAPC, Galician shearwaters were mainly assigned to Atlantic populations, with a relatively high membership probability (90.2%, see Fig 5a–5c). Only a few individuals were assigned to Selvagens (*n* = 8) and Mediterranean (*n* = 2) populations. Note that up to four individuals may be misclassified according to the error rate in the DAPC with the reference areas (see above). The three Galician colonies and the two sexes showed similar discriminant loadings (P>0.25).

When we used specific populations as reference samples in GeneClass2 to assign the origin of Galician founders (Fig 5d), half (51.4%) of the Galician shearwaters showed low assignment probabilities (<50%) and most samples were assigned to Atlantic populations (Desertas 41.7%; Azores 20.4%; Berlengas 13.6%). The assignment probabilities to each of the source populations did not differ among Galician colonies (P>0.23 in all cases) nor sexes (P>0.10), except that females had a higher probability of Canarias assignment than males (see Fig 5a; F1, 99 = 4.51 P = 0.035).

### Gene flow among Atlantic colonies

Most individuals sampled in source colonies originated in their own colony but recent gene flow among the Atlantic colonies estimated with the BayesAss analysis, is not negligible. This is especially true for Berlenga, Azores and Desertas colonies, where about 30% of the individuals in one generation are migrants from other colonies, mainly from Canarias. Immigration seemed to be lower in the Canarias and Selvagens colonies, where migrants from other colonies in one generation were about 10 and 5% respectively.

### Morphology

**Fig 6:**
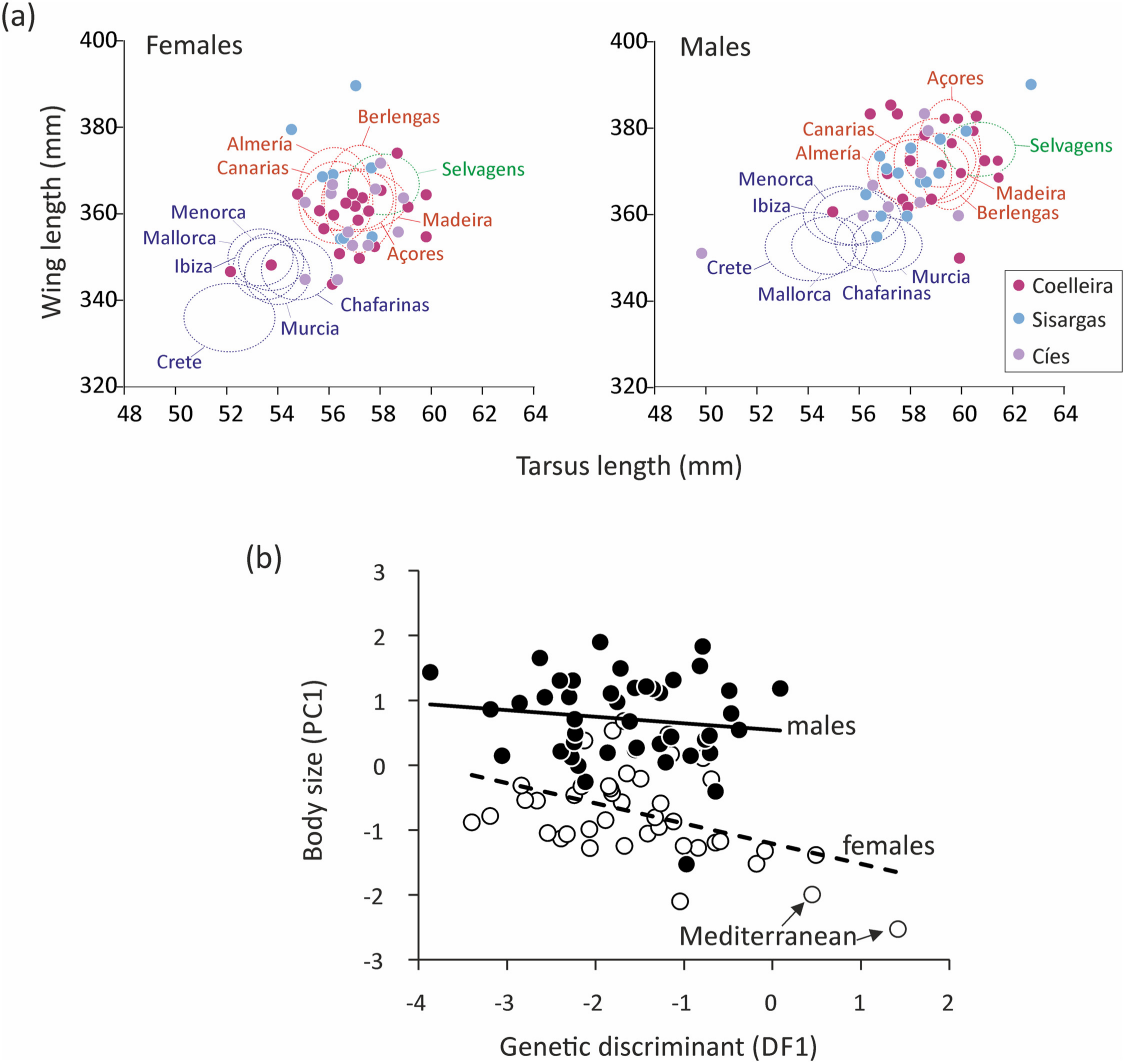
Morphometric comparison of Galician Cory´s shearwaters with those from populations elsewhere. (a) Bivariate plot of wing length against tarsus length comparing the measurements of Cory’s shearwaters sampled in Galicia (Cíes, Sisargas and Coelleira) with the average values of Atlantic (red ovals) and Mediterranean (blue ovals) populations. Ovals are centered on the mean and the length of their axes is equal to one standard deviation. (b) Relationship between body size estimated as the first principal component (PC1) of a PCA analysis and the loadings in the first discriminant function (DF1) of the DAPC performed on microsatellite data (see Fig 5). Arrows indicate the two females assigned to the Mediterranean genetic cluster by the DAPC.

The graphical comparison using wing chord and tarsus length measurements (Fig 6a) showed that the birds captured in the newly formed colonies of Galicia grouped mostly with those from the Atlantic colonies. Nonetheless, the smaller individuals in both sexes were within the mean ± 1sd interval of the Mediterranean populations used for reference. The size of Cory’s shearwaters was similar in the three Galician colonies (females: F_2,36_ = 0.24; P = 0.785; males: F_2,42_ = 1.79; P = 0.180). However the PC1 scores of one male and two females were considerably smaller than the rest (Fig 6b).

We explored the degree of agreement between morphological and genetic assignments by means of the correlation between the PC1 scores and the first genetic discriminant function DF1 of the Discriminant Analysis of Principal Components (DAPC) on microsatellite data, and found that it was significant (F_1,82_ = 8.47, P = 0.005) and dependent on sex; Sex: F_1,82_ = 100.15 P = 0.001). The relationship was stronger in females, mainly due to the two females assigned to the Mediterranean genetic cluster (Fig 5b). Nevertheless, the interaction between DF1 and sex was not significant (F_1,81_ = 1.89, P = 0.17) neither was the interaction between DF2 and sex (P>0.54).

### Cory´s shearwaters offshore Galicia according to telemetry data

**Fig 7:**
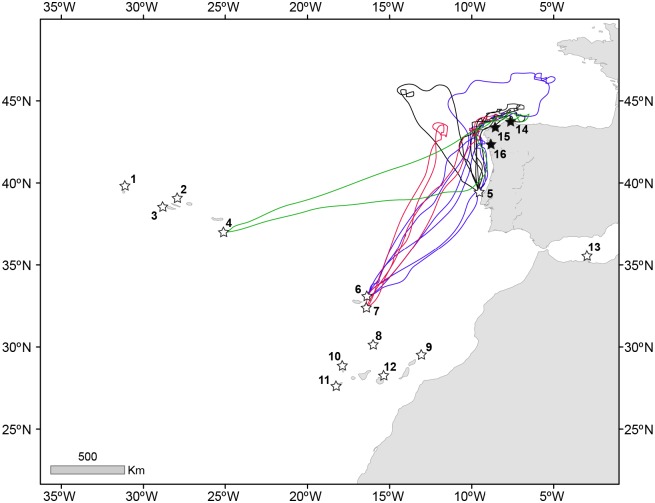
Individual tracks of the 11 Cory´s shearwaters that visited Galician waters to forage during the breeding season. White stars correspond to the main breeding colonies where tracking studies have been conducted. Telemetry data was collected on the islands of (1) Corvo, (2) Graciosa, (3) Faial, (4) Santa Maria, (5) Berlenga, (6) Porto Santo, (7) Deserta, (8) Selvagem Grande, (9) Alegranza, (10) La Palma, (11) El Hierro, (12) Gran Canaria and, (13) Chafarinas. Black stars represent the three newly established breeding areas of (14) Coelleira, (15) Sisargas and (16) Cíes in Galicia. Four individuals from Berlenga (black), three from Porto Santo (blue) and two from Deserta (red) and one from Santa Maria (green) foraged off Galicia. See Table 3 for further details.

From a total of 480 individual Cory’s shearwaters tracked in 13 breeding locations of the Atlantic Ocean and Mediterranean Sea since 2004, only 11 birds (2.1%) from four colonies targeted the waters offshore Galicia (Table 3 and Fig 7). To reach Galicia, the bird from Santa Maria (Azores) travelled 1745 km from their colony, while birds from Deserta and Porto Santo (Madeira) travelled 1423 ± 36 and 1498 ± 33 km, respectively. Birds from Berlenga, the closest breeding colony, travelled 367 ± 34 km to forage off Galicia.

**Table 3:** Tracking data collected during the breeding period (from pre-laying to chick-rearing) in different colonies of Cory’s shearwaters of the Atlantic Ocean and the Mediterranean Sea.

Study area (Archipelago)	Island	Period	N	Off Galicia	Years tracked	References
**Azores**	*Corvo*	*2004*, *2007*, *2010*	*104*	*0*		*[45, 47, 48]*
	*Graciosa*	*2006*	*14*	*0*		*[45, 47, 48]*
	*Faial*	*2006–2007*	*12*	*0*		*[45, 47, 48]*
	*Santa Maria*	*2005*, *2007*	*12*	*1*	*2007*	*[45, 47, 48]*
**Continental Portugal**	*Berlenga*	*2005–2015*	*145*	*4*	*2006*, *2011*	*[45, 48, 49]*
**Madeira**	*Porto Santo*	*2011–2014*	*28*	*3*	*2011*, *2012*	*own unpublished data*
**Desertas**	*Deserta Grande*	*2006*	*16*	*2*	*2006*	*[45]*
**Selvagens**	*Selvagem Grande*	*2008*, *2010–2011*	*41*	*0*		*[45, 48, 50]*
**Canarias**	*Alegranza*	*2006*, *2010*	*46*	*0*		*[50]*
	*El Hierro*	*2007*	*10*	*0*		*[50]*
	*La Palma*	*2007*	*10*	*0*		*[50]*
	*Gran Canaria*	*2005*, *2011*	*23*	*0*		*[50]*
**Mediterranean**	*Chafarinas*	*2001*, *2011*	*19*	*0*		*[51, 52]*
**Total**			*480*	*10*		

N = sample size (total number of individuals tracked). See Fig 4 for the location of breeding colonies.

Cory’s shearwaters foraged off Galicia both during incubation (birds breeding on Santa Maria, Berlenga and Deserta Grande) and chick-rearing (Berlenga and Porto Santo). All tracked individuals were successful breeders and usually visited the colony surroundings of other populations of the species. We didn’t notice any specific trend over the years on the number and provenance of the birds visiting Galician waters, though the sample size of tracked individuals (N = 10) is rather small to be conclusive on this topic.

## Discussion

The recent colonization of Galicia by Cory´s shearwaters was undertaken by a relatively large and admixed group of prospectors. Several lines of evidence add up to the idea that the new Cory´s shearwater populations in Galicia are composed by individuals from different sources; hence, they do not support the predictions of a dispersal hypothesis from a single colony but rather agree with predictions from island metapopulation models [38]. Moreover, distance does not appear as the main factor determining connectivity and colonization in this species.

### Establishment and early progress of Galicia colonies

During our study, more than two hundred Cory’s shearwaters were visiting the Galician colonies as indicated by the maximum number of individuals in the evening rafts assembling close to the colonies (229 birds in 2013). Both the number of apparently occupied burrows and breeding pairs increased from 2009 to 2013. We recaptured 22% of the banded adults and none of these moved to another island, which suggests that each locality may hold a distinct population of founders. Based on the size of the largest raft, founder populations may comprise around 50–70 (Cíes and Coelleira) and 150 (Sisargas) individuals, which implies that the number of active burrows grossly underestimated the size of the founder populations. This is likely an indication that during colony establishment a relatively large proportion of the birds joining the rafts do not hold a burrow. Interestingly, the same pattern was observed during the early years of the first American colony of Manx shearwaters [15]. In the three localities only a small proportion of burrows were occupied by breeders, and very few chicks were produced, suggesting that the majority of burrows were occupied by prospectors and young breeders [15]. Overall, these results suggest that the Galician colonies have been visited by a large number of prospectors, some of which progressively joined the breeding population in the following years.

Most Cory’s shearwaters breed for first time at age of six to nine years [53, 54], but since the annual productivity of the Galician colonies is currently very low (overall, four to seven chicks per year in 2009–2013), most, if not all, adults captured in Galician colonies were probably born somewhere else. An interesting question is when did the shearwaters started to visit the Galician colonies. The monitoring of Cory´s shearwaters in Galicia started in 2008 (Cíes and Coelleira), and the bird we found in May 2007 in Cíes, while censusing European shags, is the first known breeding record of Cory´s shearwaters in Galicia. Current Cory´s breeding grounds at Cíes and Sisargas have been surveyed intensively for other seabirds from the early 1990’s, including night sampling, so an unnoticed breeding colony there before 2007 is unlikely. Coelleira was a less frequently visited island, and by 2005 we were informed by the lighthouse keeper about shearwaters calling occasionally at night, but only three eggs were found in 2009. Thus, our assumption that Cory’s shearwaters founded these new colonies at the Galician coast during the mid 2000’s seems quite reasonable. Nevertheless, due to their fosorial and nocturnal habits, the possibility of earlier breeding attempts by Cory´s shearwaters in these or other insular localities of Northwestern Iberia cannot be entirely discarded. The three Galician colonies represent a northward shift of 400 km (ca. 5 latitudinal degrees) for the species range in the Atlantic, and the nearest breeding colony of Berlengas is known to exist since at least the 1870’s [55]. There is a record of a colonization attempt by a few Mediterranean birds (*C*. *d*. *diomedea*) in Arcachon (France) in the mid 2000’s [56] but it apparently failed [57]. Thus, the Galician colonies can be considered as true colony foundations, a rare event in Atlantic Cory´s shearwaters and in most seabird species. The northward shift in the Cory´s shearwater distribution is in accordance with the observed and predicted northward shift of fish and zooplankton populations in the North Atlantic. These climate-driven changes in trophic interactions in the marine food webs of the North Atlantic can affect seabird population dynamics [58].

### Genetic diversity and private alleles

Galician populations show a high genetic diversity, similar or even higher than other historical and large populations. Moreover, the mean number of private alleles per locus in the three Galician colonies exceeds that from other historical Atlantic colonies, including Berlengas, the nearest known breeding colony. Private alleles (i.e. alleles that are not found in other sampled populations) are useful for elucidating genetic diversity and relationships among populations. Assessments of the number of alleles private to different combinations of populations were also consistent with the results for individual populations. Indeed, if we exclude Selvagens, Galicia had more private alleles than the pool of sampled Atlantic populations. The private allelic richness in Galicia-Atlantic and Galicia-Selvagens pairwise combinations was higher than the pair Selvagens-Atlantic, suggesting that an admixture pool of individuals is probably participating in the colonization of Galicia. Ancestral alleles or large historical population size may explain the higher number of private alleles found in the Selvagens sample. These results argue against a founder effect resulting in loss of genetic diversity, as expected if the new populations were started by a few members of one original population [59].

### Population structure

All analyses on population structure (factorial correspondence analysis, population-level tree and individual-based clustering), revealed two main genetic demes, which broadly corresponded to the Atlantic and the Mediterranean range of the species. The differentiation between these two ocean basins has previously been reported based on mtDNA [42, 60] and on microsatellites [44]. However, under this hypothesis, a small proportion of individuals from all colonies, including Galicia, were considered to be either highly admixed, or assigned to a cluster other than their actual geographic origin. Moreover, we found some genetic differentiation within the Atlantic colonies. This was also suggested in a previous study [44] albeit separating Berlenga and Canarias colonies from the Selvagens and Azores clade, whereas in our study, Selvagens was the only divergent clade within the Atlantic cluster. Sampling may explain the difference with previous findings, because in our study individuals from Canarias were not confirmed breeders, but leaving aside this and to be highlighted, both studies suggest a spatial genetic structure within the Atlantic colonies and that distance is not the solely factor shaping population structure. We found reduced immigration in Selvagens, but in other Atlantic colonies immigration seems relatively important, which may homogenize their genetic structure. In seabirds, even low rates of immigration may be enough to result in low genetic structuring [32]. Interestingly, morphometric comparisons of Berlenga and Selvagem Grande populations, detected significant differences in all characters measured (including eggs) except for wing-length [60, 61], thus evidence suggests that individuals from Selvagens are distinct from most other Atlantic populations. However, if we are to fully understand the population structure and dispersal patterns within the Atlantic subspecies, further genetic and ecological studies that include a wider array of Atlantic colonies should be carried out.

### Origin of the founders

Bayesian assignment and discriminant analysis of principal components showed that only a few individuals could be confidently assigned to single source populations suggesting that birds settling in the Galician colonies probably originated from a genetic pool larger than the one we sampled. Most individuals assigned with a high probability to Atlantic populations, were mainly assigned to Desertas, Azores and Berlengas, but interestingly some were assigned to Selvagens and a few to Mediterranean colonies. Admixture in founder populations was further supported by the clear assignment of some of the individuals captured in Galicia to Selvagens, the only divergent clade within the Atlantic (see above).

The genetic assignment of a few individuals to the Mediterranean clade may be questionable. First, our analyses of population structure revealed a weak Mediterranean genetic signature in all Atlantic colonies, thus, the Mediterranean assignment may simply reflect this genetic background. Second, the few individuals assigned to the Mediterranean cluster fall within the error rate of our discriminant analyses, precluding robust conclusions. However, a remarkable result is that three of the smaller Galician birds (one male and two females) were those genetically assigned to the Mediterranean clade. Two clear geographic gradients in size have been detected within the Cory´s shearwaters range [60]. In the Atlantic, bird size increased with latitude whereas in the Mediterranean the increase in size was longitudinal, from east to west. Accordingly, the birds from Galicia overlapped with the size range of Atlantic colonies but the smaller individuals matched the dimensions of the western Mediterranean colonies. Thus, even if not conclusive our results are suggestive and future studies should further explore the presence of Mediterranean individuals in Galician colonies. There are some records of natal dispersal from Atlantic to Mediterranean colonies [44, 62, 63] and Atlantic shearwaters are known to breed in a few western Mediterranean colonies such as Almería, [60] and Chafarinas, [44, 51]). The recent colonization attempt in Arcachon, France [64] support the dispersal of Mediterranean birds into Atlantic colonies (see also [65] showing evidences of Mediterranean haplotypes in ancient samples from the Canarias Islands). Overall, these results may suggest a wide sympatric range between the Mediterranean and the Atlantic subspecies.

In conclusion, the picture that emerges from the array of genetic and morphometric analyses performed suggests that the Galician colonies have originated from an admixture of birds from several source colonies in the Atlantic, whereas the presence of Mediterranean birds cannot be entirely discarded.

### Models of colony formation

Our results suggest that a large and admixed population is settling in Galicia, in agreement with predictions from island metapopulation models. Possibly three groups of non-breeders, adding up to around 200 birds, started visiting the Galician colonies in the mid 2000’s and some of them eventually laid eggs and reproduced, thus establishing new breeding colonies. Our results do not support Berlengas, (the closest colony ca. 400 km apart) or any other of the Atlantic colonies as the single main source for the Galician founders. Indeed, several lines of evidence suggest admixture, which implies that some birds colonizing Galicia were dispersing from very distant colonies (> 1500 km). Previous studies have suggested that the species performs rare but recurrent long-distance dispersal events [44]. Interestingly, there are some records of comparable dispersal distances (> 1000 km) in colonizations by procellariids, as in the Laysan albatross [66], the light-mantled sooty albatross [67], the black-footed albatross [68], Manx shearwaters [15], sooty shearwaters [69] and Leach’s Storm petrels [70]. Thus, *apoikia*, the establishment of a colony far from the source populations, is likely a relatively common phenomenon in Cory´s shearwaters and other procellariids.

Large and admixed founder groups are likely to be much more efficient colonizers of new environments than are small single-source ones [71]. The beneficial effects of conspecific presence may have a positive influence in the probability of establishment by means of a varied array of mechanisms [72] especially in colonial species such as seabirds [73, 74]. Admixture increases the genetic diversity of populations and promotes heterosis (i.e. hybrid vigour), which can be particularly beneficial during periods of range expansion, when novel territories are colonized [8]. Long-distance colonizations undertaken by relatively large and admixed groups of colonizers can explain the low levels of genetic structure over vast areas that are characteristic of many of the most oceanic seabird species [75, 76]. Thus, the occasional formation of founder groups made of pioneers reared in a collection of distant source colonies can be a way to overcome the seabird paradox with respect to dispersal potential and realized gene flow [32]. The colonization mechanism suggested in this study for the newly established colonies of Galicia may not be limited to Cory´s shearwaters but may be relatively widespread, at least in oceanic birds such as procellariids as suggested by the development of colonization events in Manx shearwaters [15] and Laysan albatross [38]. Even if we take into consideration that due to behavioral (attraction to immigrants) and demographic (delayed maturity) constraints, new colonies in procellariids and other seabirds can take several years to establish, their ability to colonize distant localities may help them to rapidly and efficiently respond to large-scale environmental changes and extreme stochastic events [77, 78].

## Materials and Methods

This study was carried out with appropriate licensing and permissions for scientific work in restricted access natural areas and bird handling and ringing in Galicia, issued by Xunta de Galicia and the National Park of the Atlantic islands of Galicia. All sampling procedures were specifically approved as part of obtaining the field permit. The issuers of the permit did not require approval of field sampling methods by an animal ethics committee. We made all possible efforts to reduce handling times and to keep disturbance to the birds to a minimum.

### Field procedures at the Galician colonies

This study was conducted in the three breeding areas that the Cory´s shearwaters have recently established in Galicia, at the NW of the Iberian peninsula, in the Northeast Atlantic (Fig 2): the island of Coelleira (43°45’31”N, 07°37’51”W), Sisarga Grande (43°21’33”N, 08°50’28”W) and the archipelago of Cíes (42°14’04”N, 08°54’14”W). Cíes, the only locality within a protected area, is part of the National Park of the Atlantic islands of Galicia. Terrestrial carnivores, a severe threat to ground nesting seabirds, have been introduced to Cíes (American mink and feral cat) and Sisargas (feral cat). Cory´s shearwater burrows are located in westward oriented cliffs of moderate to steep slope and facing the open sea. It is unlikely that any of these colonies were started before the mid 2000’s, especially in Cíes and Sisargas, as these islands have been intensively surveyed for other seabirds since the mid 1990’s. Coelleira is a less frequently visited island but when the colony was discovered (2008) it was very small. The systematic study of the onset and early progress of these new breeding colonies extended from 2008 to 2014. Thus, during 2009 to 2013 two or three surveys were conducted in each locality annually in search of apparently occupied burrows (AOBs). In the surveys, all the AOB´s detected were individually tagged and signs of shearwater occupation recorded. Additional field work was conducted in 2008 (Cíes and Coelleira) and 2014 (Cíes). Each survey consisted of two separate searches, both at night and in daylight. Thus, in all occasions, the areas were the shearwaters called and landed were searched intensively at night while the birds were arriving and are most easily observed. Daylight searches involved the examination of all tagged sites as well as potential unmarked burrows with the aid of sound recordings of both male and female calls. Breeding was confirmed when either eggs or chicks were observed. All adults were captured at night by hand and most were found in suitable breeding places. All captured individuals were marked with a metal ring, weighed and measured: bill length (exposed culmen), bill depth at nostril, head, tarsus and wing. From each captured bird, a small blood sample (ca. 100 μl) was also taken from the leg vein. We collected blood in a capillary tube and transferred it into a 2 ml tube with ethanol. Samples were stored at room temperature before genetic analysis. During the evening prior to the night searches we estimated the size of any Cory’s shearwater rafts assembling in the marine area that was visible from the colony. This area was surveyed during the two hours before sunset by two observers using binoculars. Additionally, two boat censuses were conducted yearly off Cíes with the same purpose.

### Sample collection of candidate founder populations

To assign possible source populations, we genetically analyzed 174 blood samples of individuals from five populations representing the entire range of the Cory´s shearwater in the Atlantic: Berlengas (mainland Portugal), Tenerife (Canarias), Selvagen Grande (Selvagens), Deserta Grande (Desertas) and Corvo (Azores); and three from the Western Mediterranean: illa de l’Aire (Menorca), Pantaleu (Mallorca) and Habibas (Tunisia) (see Table 1); we additionally included as control samples in some analyses, four individuals from Chafarinas, where the Atlantic and the Mediterranean taxa breed sympatrically [44, 51]. Except in Tenerife, where the samples were obtained from injured fledglings and juveniles taken to a rehab center, all the individuals sampled were adults attending the colonies. Colonies were visited during the breeding period and blood from the breeding adults (ca. 25 μl) was sampled from the leg vein. Samples were stored into a 2 ml tube with ethanol at room temperature before genetic analysis

#### DNA extraction and amplification

Total DNA was isolated from blood samples by an overnight incubation at 55°C in SET buffer with 30 μl SDS 10% and 2.5 units/ml of proteinase K followed by a standard phenol/chloroform protocol [79]. DNA was resuspended in TE buffer [79]. We analyzed 10 microsatellite loci, 6 previously design for the Balearic shearwater *Puffinus mauretanicus* [80] and polymorphic in this species [44] and 4 specifically designed for this species [81]. Amplification reactions were performed in a total volume of 10 μl with 0.4μM of each primer (fluorescence labeled with VIC, NED6, FAM, PET and FAM), 0.2 mM dNTP, 1x Taq buffer, 1 U of Taq DNA polymerase (Bioline), 2–3 mM of MgCl_2_ (depending on the primer) and 1–2 μl of template DNA. The thermocycling conditions were 94°C for 2 min, followed by 34 cycles of 95°C for 30 seconds 50°C–60°C for 30 seconds and 72°C for 30 seconds, with a final extension of 72°C for 5 min. Reactions were loaded together and the length of the DNA fragments were analyzed directly from PCR product using an ABI 3100 automated sequencer (Applied Biosystems, Warrington, UK) and software ABI GeneMapper v. 3.7 and finally rechecked by eye.

We also assigned sex to sampled individuals by PCR amplification of the CHD genes [82, 83]. Blood was boiled in 100mM NaOH for 10 minutes at 100C before being added to the PCR reaction. PCR protocols were modified from Fridolfsson and Ellegren [84] using the primer set 2550F–2718R, and products were run out on a 3% agarose gel stained with ethidium bromide. Gels were scored as males having a single band and females having two bands.

#### Genetic diversity and private alleles

We measured the mean number of alleles per locus, as well as observed and unbiased expected heterozygosity [85] using the software Genetix v. 4.05 [86]. With the same software, we also calculated the inbreeding coefficient (F_is_) and tested for deviations from the Hardy-Weinberg equilibrium. Using permutations (>1000) we tested for the occurrence of non-random associations of pairs of loci (linkage disequilibrium). We evaluated the mean number of private alleles per locus in individual populations and in various sets of populations grouped according to major breeding areas [87]. Since the number of private alleles (i.e.: alleles that are not found in other sampled populations) depends severely on sample size, we used a rarefaction approach as implemented in the computing program ADZE [87]. Under a single source scenario, we expected a low number of private alleles in founder populations compared to source populations. Additionally, we also applied the rarefaction approach for counting alleles private to all pair combinations of the major breeding areas. Private allelic richness for a pair combination estimates the number of distinct alleles private to a group of populations and found in all populations in the group, thus indicative of shared ancestry.

### Spatial genetic structure

We calculated F_ST_ with sample size correction as the basis for generating a neighbor-joining (NJ) tree based on population allele frequencies using the program Poptreew [88]. The tree was midpoint rooted, and levels of confidence in tree nodes were calculated from 10,000 bootstrap replications. F_ST_ values were taken as representative of genetic distances between populations and the significance of the correlation coefficient between genetic and geographic distances was estimated using a Mantel test implemented in Poptools [89]. To visualize the patterns of differentiation among populations we performed a factorial correspondence analysis of multilocus scores (MCA) using Genetix v. 4.05 (10 loci, 3 factors). Conventionally, the first axis has a larger contribution to total inertia and usually displays the differentiation between species or subspecies.

We assessed population differentiation among colonies by Bayesian assignment techniques using the software Structure 2.3.4 [90]. We identified clusters (K) of genetically similar individuals from multilocus genotypes without prior knowledge of their population affinities, using the *F* model of correlation in allele frequencies across clusters [91] and allowing admixture among populations. A series of preliminary runs (three independent runs of 100 000 iterations, following a burn-in period of 10 000, for each value of K from 1 to 12 clusters) revealed that the true value of K ranged between 2 to 3 clusters, as indicated by ΔK [92] calculated using the software Structure Harvester v 0.6.1 [93]. Thus, for a more detailed analysis, we ran ten replicates using 800 000 iterations, with a burn in of 400 000, of each K from 1 to 4 clusters, using the same parameters as the pilot study. We obtained the population and individual mean across replicates of the cluster membership coefficients of each individual and population with Clumpp v. 1.1.1 using *FullSearch* algorithm [94].

### Population genetic assignment

We assigned Galician samples to the most likely genetic source location using the Bayesian assignment test method in Geneclass2 [95] and the Rannala and Mountain computation criteria [96]. We first assigned samples to the most probable population between Mediterranean and Atlantic area. In a second run, we used the nine populations as reference sample. We also estimated the assignment probability by 10,000 Montecarlo simulations using Paetkau et al. (2004) algorithm [97].

We additionally assigned Galician shearwaters to reference populations by means of a Discriminant Analysis of Principal Components (DAPC, [98]) using the adegenet package in R [99]. This method first transformed genetic data of reference individuals using principal component analysis and performs a discriminant analysis on these uncorrelated components. The data from individuals to be assigned is centered and scaled using the reference individuals, and discriminant functions are used to predict the position of individuals.

### Contemporary gene flow among Atlantic colonies

Since Galician colonies were grouped within an Atlantic genetic deme, and to help disentangling connectivity patterns, we explored the contemporary gene flow (i.e. during the past one-to-three generations) between the Atlantic colonies using the Bayesian assignment algorithm implemented in BayesAss [100]. A preliminary BayesAss analysis was conducted with the microsatellite data set using the default delta values for allelic frequency, migration rate, and inbreeding. Subsequent analyses incorporated different delta values to ensure that proposed changes between chains at the end of the run were between 20% and 60% of the total chain length [101]. Once the delta values (ΔA = 0.70, Δm = 0.85, and ΔF = 0.85) were almost within the accepted proportion of proposed changes (A = 50%, m = 59%, and F = 66%), analyses were conducted three additional times (50 million iterations, ten million burn-in, and sampling frequency of 2,000) with different random seeds. Even with ΔF of 0.99 we could not decrease the acceptance rates. All parameter estimates converged.

### Morphological analyses and telemetry data

The morphological analyses of the Galician birds were conducted on a total of 86 sexed adults (45 females and 41 males) from the three colonies: Coelleira (N = 43), Sisargas (N = 22) and Cíes (N = 21). We used a principal components analysis (PCA) for each sex separately on the correlation matrix with four variables (bill depth, bill length, tarsus and wing) standardized to zero mean and unit variance. The normality of distributions was assessed graphically by plotting the cumulative frequency distribution of the samples. In both sexes the first two components explained more than 65% of the observed variability (females: PC1 = 45.4%, PC2 = 22.4%; males: PC1 = 45.7%, PC2 = 25.0%). The PC1 values were used as indicating overall size. Morphometric data from colonies elsewhere was taken from [38] (Table 1) and [61].

We used telemetry data (compass- and GPS-loggers and PTT transmitters) to assess the geographic origin of the birds that visit the coastal and offshore waters of Galicia during the breeding season. The telemetry data corresponds to 480 individual Cory’s shearwaters tracked in 13 breeding locations throughout the Atlantic and the Mediterranean (Chafarinas), and was mainly obtained from the literature (see Table 3).
